# Factors shaping the COVID-19 epidemic curve: a multi-country analysis

**DOI:** 10.1186/s12879-021-06714-3

**Published:** 2021-10-02

**Authors:** Su Yeon Jang, Laith Hussain-Alkhateeb, Tatiana Rivera Ramirez, Ahmed Asa’ad Al-Aghbari, Dhia Joseph Chackalackal, Rocio Cardenas-Sanchez, Maria Angelica Carrillo, In-Hwan Oh, Eduardo Andrés Alfonso-Sierra, Pia Oechsner, Brian Kibiwott Kirui, Martin Anto, Sonia Diaz-Monsalve, Axel Kroeger

**Affiliations:** 1 Department of Preventive Medicine, Kyung Hee University School of Medicine, Seoul, Korea; 2 Global Health, School of Public Health and Community Medicine, Institute of Medicine, University of Gothenburg, Medicinaregatan 18A, 41390 Gothenburg, Sweden; 3 Centre for Medicine and Society, Albert-Ludwigs-University Freiburg, Bismarckallee (3’d floor), 79089 Freiburg, Germany; 4 Laboratorio de Salud Pública, Instituto Departamental de Salud-IDS, Norte de Santander, Colombia; 5 Grupo GIGA, Universidad Francisco de Paula Santander, San José de Cúcuta, Colombia; 6 Angewandte Gesundheitswissenschaft, Ravensburg-Weingarten University, Weingarten, Germany; 7 Present Address: Bangalore, India

**Keywords:** Coronavirus disease 2019, SARS-CoV-2, Segemented Time-series, Lockdown, COVID-19, Stringency index, Human mobility

## Abstract

**Background:**

Lockdown measures are the backbone of containment measures for the COVID-19 pandemic both in high-income countries (HICs) and low- and middle-income countries (LMICs). However, in view of the inevitably-occurring second and third global covid-19 wave, assessing the success and impact of containment measures on the epidemic curve of COVID-19 and people’s compliance with such measures is crucial for more effective policies. To determine the containment measures influencing the COVID-19 epidemic curve in nine targeted countries across high-, middle-, and low-income nations.

**Methods:**

Four HICs (Germany, Sweden, Italy, and South Korea) and five LMICs (Mexico, Colombia, India, Nigeria, and Nepal) were selected to assess the association using interrupted time series analysis of daily case numbers and deaths of COVID-19 considering the following factors: The “stringency index (SI)” indicating how tight the containment measures were implemented in each country; and the level of compliance with the prescribed measures using human mobility data. Additionally, a scoping review was conducted to contextualize the findings.

**Results:**

Most countries implemented quite rigorous lockdown measures, particularly the LMICs (India, Nepal, and Colombia) following the model of HICs (Germany and Italy). Exceptions were Sweden and South Korea, which opted for different strategies. The compliance with the restrictions—measured as mobility related to home office, restraining from leisure activities, non-use of local transport and others—was generally good, except in Sweden and South Korea where the restrictions were limited. The endemic curves and time-series analysis showed that the containment measures were successful in HICs but not in LMICs.

**Conclusion:**

The imposed lockdown measures are alarming, particularly in resource-constrained settings where such measures are independent of the population segment, which drives the virus transmission. Methods for examining people’s movements or hardships that are caused by covid- no work, no food situation are inequitable. Novel and context-adapted approach of dealing with the COVID-19 crisis are therefore crucial.

**Supplementary Information:**

The online version contains supplementary material available at 10.1186/s12879-021-06714-3.

## Background

Soon after the start of the COVID-19 pandemic in China, many countries followed the Chinese example to put emphasis on transmission control through quarantine of infected persons, the isolation of contacts, and then the lockdown of the entire population [1, 2]. The theoretical background was based on a mathematical prediction model about the expected epidemic curve showing that without transmission control, the curve would be very high and stiff but with “non-pharmaceutical interventions (NPIs)”, it would be prolonged but with lower case numbers and thus manageable by the health services [3]. Strict lockdown measures were particularly undertaken in the high-income countries (HICs) of Europe and then carried forward by low-and middle-income countries (LMICs), often leading to economic recession, human suffering and public unrest due to job and income loss [4, 5]. However, there were some different approaches with less stringent lockdown policies such as in Sweden and South Korea [6]. This provides an opportunity to analyse counter-measures and the level of compliance to such measures leading to the highest impact on the shape of the epidemic curve, five months after the start of the pandemic. Additionally, it enables to investigate if the real-life experience in different countries confirms the previously defined prediction model [3], and intends to show “when” and “how much” containment measures are successful in low and high income countries which will help to take up evidence based policy decision according to the local context.

## Methods

### Study countries

Four HICs (Germany, Sweden, Italy, and South Korea), two better-off MICs (Mexico and Colombia), and three LICs with lower economic power (India, Nigeria, and Nepal) were selected in order to reflect the measures and impact of the covid-19 pandemic in countries with different levels of wealth, size and population densities. Academic contacts in these countries facilitated the data collection. Table 1 shows that large (India, Nigeria, and Mexico) and small countries (Sweden and Nepal) with high (South Korea and India) and low population densities (Sweden and Colombia) were included. The percentage of children within the total population was less in countries with a high Human Development Index (European countries and South Korea) and more if the index was lower (Nigeria, Nepal, and India). Table 2 shows that countries with a high Gross Domestic Product (GDP) have a low informal economic sector and the LMICs have a high proportion of “self-employed” or “vulnerable employment” who will suffer most by the lockdown measures. The only exception was South Korea, with a high GDP but yet a considerable informal economic sector, due to its recent history of moving from a LMIC to a HIC.

**Table 1:** Demographic, geographic, and developmental characteristics of the 9 target countries

Country	Total population (thousands)	Population density (persons per km^2^)	Percentage of children (0–14 year) (%)	Human Development Index (value)
Germany	83,517	240	14	0.939
Italy	60,550	206	13	0.883
Sweden	10,036	25	18	0.937
South Korea	51,225	527	13	0.906
Colombia	50,339	45	22	0.761
Mexico	127,576	66	26	0.767
Nigeria	200,964	221	45	0.534
India	1,366,418	460	26	0.647
Nepal	28,609	200	29	0.579

Data Sources: United Nations: World Population Prospects 2019 (online: https://population.un.org/wpp/Download/Standard/Population/); United Nations Development Programme: Human Development Report 2019, p. 304 (online: http://hdr.undp.org/sites/default/files/hdr2019.pdf)

**Table 2:** Selected economic indicators of the nine target countries (World Bank, World Development Indicator, 2018)

Name	Sweden	Germany	South Korea	Italy	Mexico	Colombia	India	Nigeria	Nepal
GDP per capita, PPP (constant 2011 international $)	47,718	45,936	36,777	35,828	18,134	13,321	6888	5316	2741
Poverty headcount ratio at $3.20 a day (2011 PPP) (% of population)	0.3%	0.2%	0.5%	1.8%	6.6%	10.9%	60.4%		50.9%
Self-employed, total (% of total employment) (modeled ILO estimate)	9.6%	9.9%	25.1%	22.9%	31.6%	51.4%	76.5%	81.5%	79.8%
Vulnerable employment, total (% of total employment) (modeled ILO estimate)	6.1%	5.7%	19.2%	16.8%	26.8%	47.2%	74.5%	77.7%	78.7%
Informal employment (% of total non-agricultural employment)						57.2%	80.3%		77.6%

Source: World Development Indicators (WDI), compiled by the World Bank from officially recognized international sources. https://data.worldbank.org/indicator?tab=all

"Employment in the informal economy as a percentage of total non-agricultural employment. It basically includes all jobs in unregistered and/or small-scale private unincorporated enterprises that produce goods or services meant for sale or barter. Self-employed street vendors, taxi drivers and home-base workers, regardless of size, are all considered enterprises. However, agricultural and related activities, households producing goods exclusively for their own use (e.g. subsistence farming, domestic housework, care work, and employment of paid domestic workers), and volunteer services rendered to the community are excluded"

"Self-employed workers are those workers who, working on their own account or with one or a few partners or in cooperative, hold the type of jobs defined as a self-employment job (i.e., jobs where the remuneration is directly dependent upon the profits derived from the goods and services produced). Self-employed workers include four sub-categories of employers, own-account workers, members of producers’ cooperatives, and contributing family workers"

"Vulnerable employment is contributing family workers and own-account workers as a percentage of total employment"

"Poverty headcount ratio at $3.20 a day is the percentage of the population living on less than $3.20 a day at 2011 international prices.”

“Proportion of employed people who live on less than $3.20 (in purchasing power parity terms) a day, expressed as a percentage of the total employed population ages 15 and older.” ILO (2019). ILOSTAT database. www.ilo.org/ilostat

“Percentage of the population at risk of suffering multiple deprivations—that is, those with a deprivation score of 20–33 percent.” Source: Human Development Report Office (HDRO) calculations, based on data on household deprivations in health, education, and standard of living from various household surveys. Latest data for Mexico, Colombia, India, Nepal from year 2016, Nigeria year 2017. http://hdr.undp.org/en/indicators/142506

### Scoping review of the literature

To complement our findings, a scoping review of the scientific literature was conducted with the following question: “What measures can influence the COVID-19 epidemic curve among the nine targeted countries?” The scoping review, as an ideal approach to determine the scope of a body literature on covid-19 while examining emerging evidence in a short period of time, was the prime choice in this study.

On May 9, 2020, a search was conducted in online databases, PubMed and Cochrane library with key search terms such as “COVID-19”, “measures”, and “factors”. The inclusion criteria were scientific articles with information on influencing factors of COVID-19 epidemic curve and public health measures adopted by nine targeted countries. Observational and intervention studies including qualitative, quantitative, or mixed methodologies, as well as scoping reviews and full text papers in English or Spanish language were included. Preprint scientific studies were included due to the urgency of the pandemic. The excluded articles were letters to the editor, opinions, guidelines, commentaries and editorials. The study selection was done independently by three researchers (TRR, MAC, and RCS). The three sets of literature were then compared. Disagreements on the inclusion or exclusion of literature were solved through discussions or by including a fourth researcher (AK). The search was carried out in three stages. First, titles were evaluated according to the inclusion and exclusion criteria. Second, the same criteria were applied to the abstract section of the articles retained in the first stage. Third, full text articles and articles without abstract availability in the previous stages were evaluated. However, after completion of the scoping review, new publications came up which will be presented in the “Discussion” Section.

### Data collection and analysis in the nine study countries

#### Measuring number of daily infections and deaths

Daily numbers of infections and deaths were collected from various existing data sources. Each country’s national confirmed and deceased cases were collected through a data hub of COVID-19 datasets [7]. Furthermore, the COVID-19 related situation on a sub-national level was analyzed. Targeted regions were: Västra Götaland (Sweden), Lombardy (Italy), Baden-Württemberg (Germany), Daegu (South Korea), Kathmandu (Nepal), Nuevo León (Mexico), Abuja (Nigeria), North Santander (Colombia), and Kerala (India). For European regions, data was obtained from the above mentioned repository [8], however, non-European areas were not accessible through the repository, and thus collected through each country’s national or regional official health service websites [9–14]. Rates of infection and deaths per 100,000 population were estimated for all nine countries based on the study period from January–May 2020 and the corresponding mid-year population size as the denominator [15].

#### Measuring timing and intensity of the lockdown

To assess the intensity of lockdown measures in the target countries, the ‘Stringency Index’ (SI) of the strictness in governmental policies was calculated using eight indicators: closure of schools, workplaces, public events, and/or of public transportation; restrictions on gatherings, internal movements, and international travels; and the quarantine requirements. Computation of the index followed the methodology described by Hale et al., which estimated the intensity of governmental measures into a scale from 1 to 100, with 100 indicating the maximum application of all indicators mentioned above [16]. The mean (SD) and percentiles (25th, 50th, 75th, and 95th) of the SI were computed for each country and sub-country. Data of government responses in our target countries was collected from the Oxford COVID-19 Government Response Tracker [17].

#### Measuring peoples` compliance with the lockdown

For the documentation of peoples’ compliance with the lockdown, data from the Google COVID-19 Community Mobility Reports was used to measure the change in human mobility [18]. In these reports, percentage of changes in visits to different places (i.e., retail and recreation, grocery and pharmacy, parks, transit stations, workplaces, and residential areas) were compared to the baseline level (i.e., the median value from January 3rd to February 6th 2020) and were estimated by aggregating the location data of Google account holders.

### Data management and analysis

#### Data management of the scoping review

**Fig. 1:**
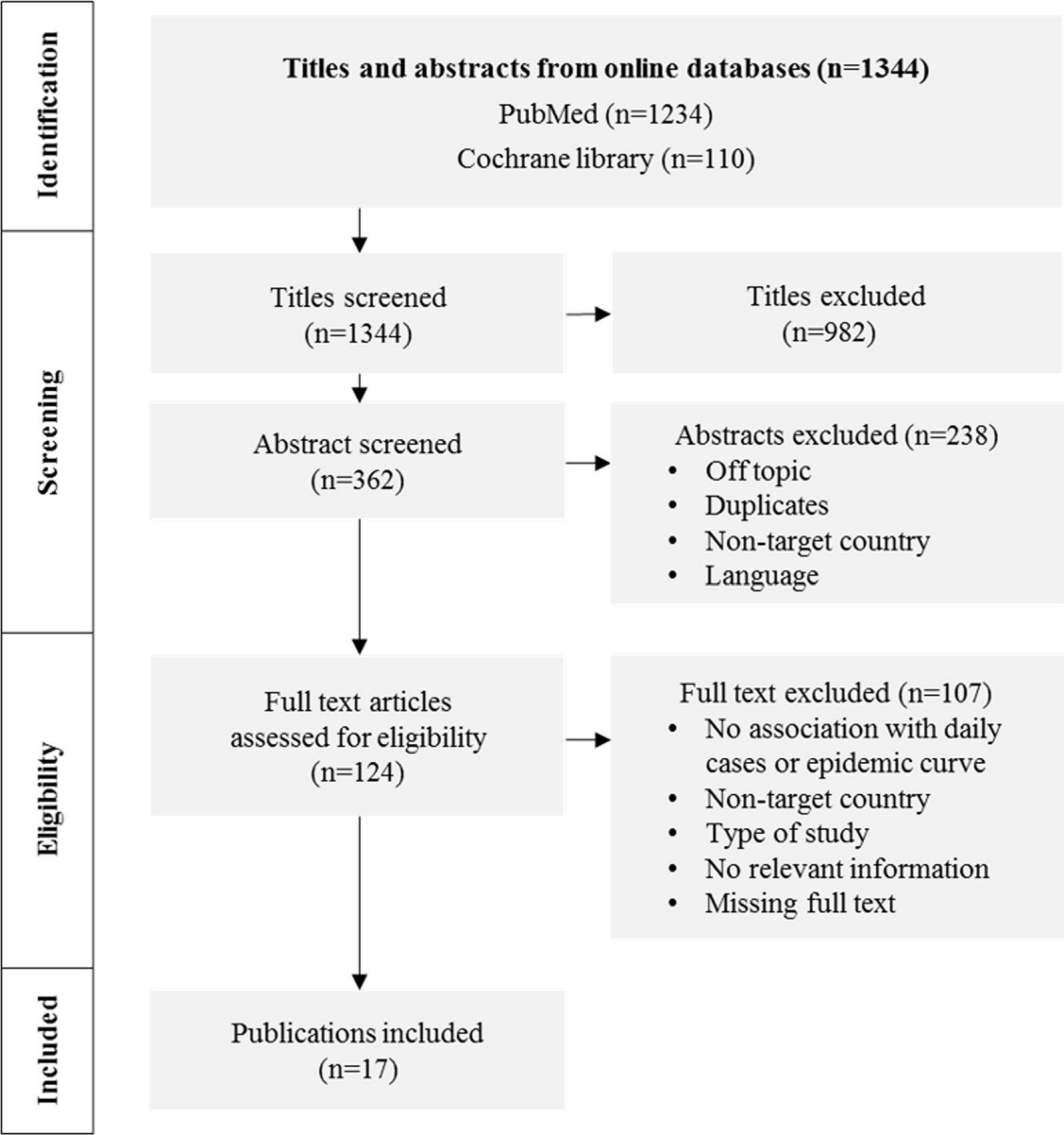
PRISMA 2009 Flow Diagram. Search results and selection process of the studies on containment measures influencing the COVID-19 epidemic curve in the nine targeted countries. Targeted countries were Sweden, Italy, Germany, South Korea, Nepal, Mexico, Nigeria, Colombia, and India

Data from the included studies in the scoping review were extracted and recorded in an Excel spreadsheet. The following information was collected for each article: Authors of the publication, country, study design, status of the publication, analysed measure (e.g., school closures or the lockdown), methodology, instruments, and results. No formal assessment of the methodological quality of the included articles was performed in this review, however, the quality of the papers was defined by the inclusion and exclusion criteria. Figure 1 shows the selection process of the papers [19]. A total of 1344 papers were initially retrieved. After the application of the inclusion and exclusion criteria, 17 publications were included for the synthesis of the review (Additional file 1). A narrative description is presented in the “Results” Section.

#### Interrupted time-series analysis

To better understand the impact of the SI on the incidence and mortality as core outcome variables, an interrupted time-series analysis of mortality and morbidity rates was conducted independently per country and sub-country [20]. The SI is the analysis predictor; in order to transform its continuous format into a meaningful intervention measure before processing the time-series analysis, the median value of SI in each country was defined as “intervention one,”. This was based on observations that the SI was able to demonstrate an impact in relation to the baseline values (both the minimum and 25th percentile) at the 50th percentile. A sensitivity analysis was performed to test this assumption; an exception was made for Sweden where the 10th percentile value was used as “intervention one” due to their relatively low stringency measures. Second and third points of interventions were defined based on 75th and 95th percentiles, respectively. In this analysis some countries managed to implement all three interventions, whereas other countries implemented one or two interventions. When two percentiles revealed small differences (< 10%), the higher percentile was used in the regression. “Baseline trend” refers to the change in rate prior to intervention one; “change at first, second, or third interventions” refers to the change in rate immediately after each intervention; “trend after each intervention” refers to the continuous change in rate after the current intervention and until the next intervention; and the “overall trend after all interventions” refers to the change in rate due to all interventions. These four trends were presented as “rates of change per 100,000 population” together with their p-value at 5% significant cut-off.

## Results

### Dynamics of the COVID-19 pandemic according to the scoping review

#### Time lag between containment measure, daily slow-down effect, and deaths

A study in Italy demonstrated that the containment measures reduced the progression of the COVID-19 epidemic [21]. The time lag between implementing measures to the reduction of COVID-19 growth rate was approximately 7–10 days. The analysis in different Italian regions showed that the earlier the measures were taken, the lower was the cumulative incidence. The importance of implementing early measures was also observed in 25 European countries: as countries with the highest mortality (Italy, Spain, and France) were late to implement national restrictions. Sweden adopted fewer restrictions compared to other neighbouring countries and suffered a higher mortality rate [22].

#### Daily growth rate in a controlled and uncontrolled situation

Various modelling studies analysed different scenarios to control the spread of COVID-19. A study assessed the effectiveness of social distancing in Italy based on the level of adherence to quarantine. They predicted a three-persons household with each level of complete, nearly-complete, medium, and no quarantine to have 7, 8, 12 and 20 secondary infections, respectively, during 14 days [23]. In large households with 6 persons, 16, 19, 29, and 43 secondary infections in 14 days respectively were predicted for each of the quarantine completion level, suggesting that a higher adherence to quarantine and a smaller household contribute to a lower number of secondary cases. In South Korea, during the outbreak, there was a positive correlation of compliance with lockdown measures and a decline in the confirmed cases [24]. Likewise, “home office” and the delay of school opening led to a marked transmission reduction.

Choi and Ki simulated the epidemic in South Korea and predicted nearly 5 million COVID-19 cases without any measures, while the lockdown could reduce the transmission rate by 90% to 99% [25–27]. The combination of different mitigation measures seems to be crucial for reducing infections and deaths [26], just as the increased compliance with the measures [28]. In Veneto, Italy, seventeen days after the lockdown strategy, 658 hospitalized cases (95% CI 618–698) could be prevented and the peak of the curve was delayed by 3 days [29]. In Italy, measures such as “red zone” (lockdown in ten towns in Lombardy) effectively contained the spread of the infection and the general lockdown had a positive effect in other regions of Italy [30].

A modelling study in India found that within 21, 42, and 60 days lockdown periods, the number of cases (378,036 infections without intervention) was reduced to 70,424 after 110 days in the 21 days lockdown-scenario, and was additionally reduced to 42,950 in the 42 days scenario, but no additional reduction by prolonged lockdown of more than 42 days [31]. Another modelling study in France showed that the isolation of individuals with no or mild symptoms was not sufficient to reduce the number of confirmed cases, however, both in France and North-Italy, a substantial case reduction could be achieved by a large-scale reduction of social interactions [32]. A study in South Korea estimated the effective reproduction number (*R*_*0*_) to be 1.5 (95% CI 1.4–1.6), the intrinsic growth rate to be 0.6 (95% CI 0.6–0.7) and the “deceleration of growth” to be 0.8 (95% CI 0.7–0.8), which indicate for sub-exponential growth dynamics of COVID-19 [33].

#### Reduction of peak prevalence, cumulative incidence, and R_0_ by containment measures

A study in Italy and Spain comparing daily percent increase of diagnosed cases, deaths, and ICU admission before and after the national lockdowns showed that before lockdown, daily percent of incidence was high in Spain (38.5% of diagnosed cases, 59.3% of deaths, 26.5% of ICU admissions) and less in Italy (21.6%, 32.8%, and 16.7% respectively), however, after the first lockdown, incidence was considerably lower in both countries (11.9%, 17.6%, and 9.6% in Spain, respectively, and 2.5%, 13.7%, and 3.7% in Italy) [34]. After the second and more restrictive lockdown, particularly in Italy, all outcomes declined (−2.0%, −0.2%, and −16.8% respectively), and so it happened in Spain (−2.7%, −1.8%, and −5.6% respectively).

A modelling study in India showed that if 50% of symptomatic cases are in quarantine within three days after developing symptoms, assuming a minimal basic reproduction number R_0_ of 1.5 before symptoms develop, the decrease of the cumulative incidence was 62% and of the peak prevalence was 89%. In contrast, when assuming that R_0_ was 4 and the infectiousness of asymptomatic cases was half compared to symptomatic cases, the estimated cumulative incidence will decrease by only 2% and the peak prevalence by 8% [35]. In another modelling study in India, lockdown measures reduced the basic reproduction number from 2.3 before the lockdown to 0.15 after the measure [36].

#### Age specific infection rates and case fatalities

A study in Germany showed that after establishing physical distancing in week 12, people aged 15–34 years played a predominant role in the spread of the disease compared to older (35–39 years) and younger age groups (10–14 years), assuming that the non-adherence to social distancing was frequent in this age group [37].

In Korea, Daegu province, the outbreak generally began in the younger age groups, but case fatalities were the highest among people aged ≥ 80 years (12.1%), followed by those aged 70–79 years (5.6%) [38]. In 66 laboratory confirmed fatal cases of COVID-19, the median age was 77 years (range, 35–93 years), and female-to-male ratio was 44:56. In South Korea, the crude case-fatality was higher among males (1.1%) compared to females (0.4%) and increased with older age [33].

#### Risk of importation and airport measures

One of the first measures implemented by the Italian government was to suspend flights from China and install in air-ports’ checkpoints with thermoscans. However, this measure appeared not to be very effective to contain the epidemic [30]. Mandal et al. found in a modelling study in India that airport screening of symptomatic arrivals will lead to a delay of 2.9 days in the predicted “average time to epidemic (days to reach a prevalence of 1000 cases)” [35]. In order to get a delay of 20 days, an additional 90% coverage in the screening of asymptomatic passengers will be needed, which is difficult to achieve, however, there are additional benefits of identifying asymptomatic arrivals rather than screening only symptomatic cases [35].

### The shape of the epidemic COVID-19 curves and determinants in 9 countries

#### Cumulative and daily infections and deaths

**Fig. 2:**
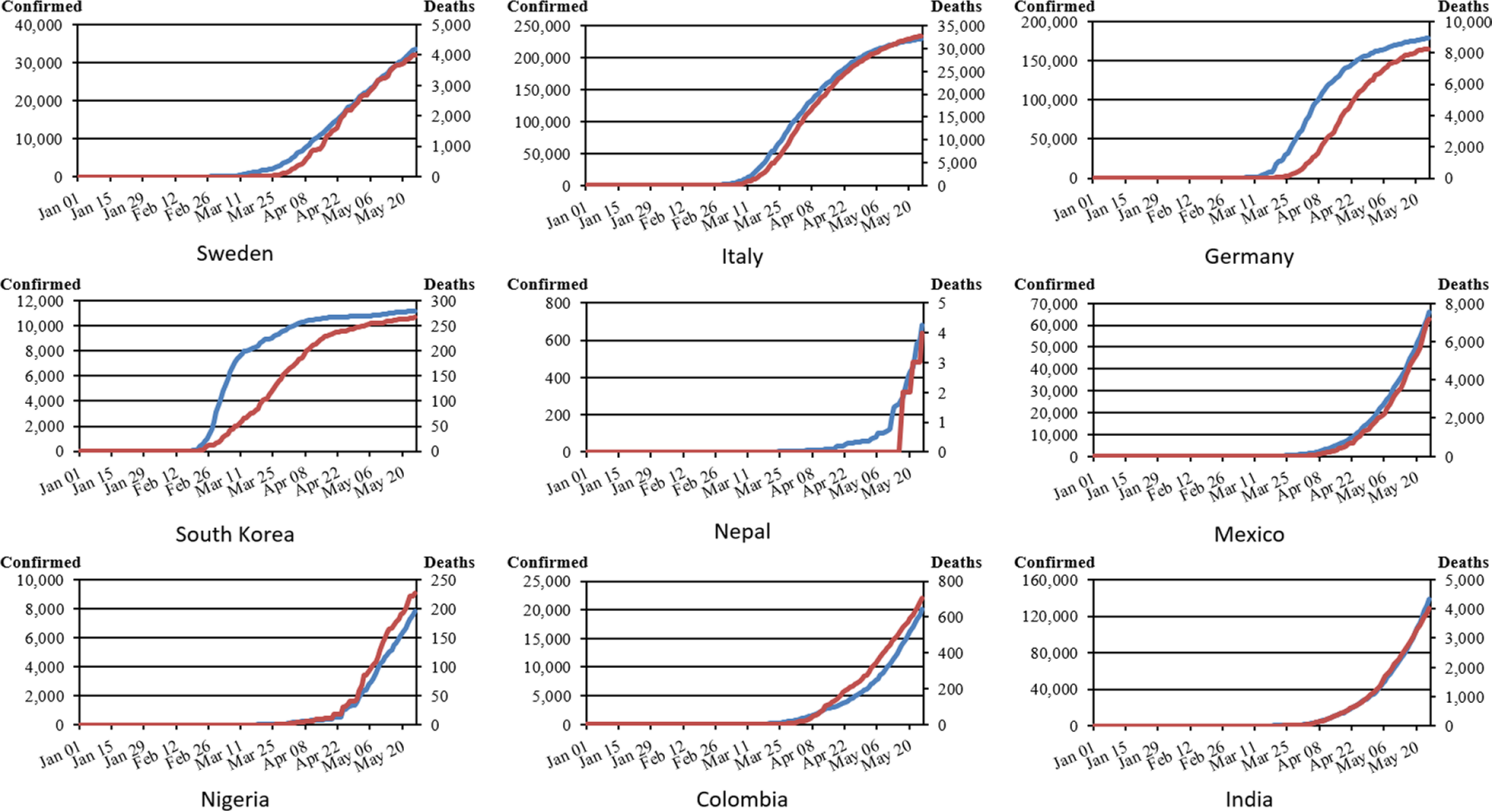
Cumulative incidence of COVID-19 cases (“cases of infection” or “cases”) and deaths. Blue = Cumulative cases of infection, Red = Cumulative deaths

Figure 2 shows the cumulative incidence of COVID-19 until the end of May 2020 (both laboratory confirmed and non-confirmed). There are mainly two types of epidemic curves; in Germany, Italy, and South Korea, a quick increase in cases can be seen followed by a slow-down of the transmission, while there was almost a linear increase in Sweden. In the LMICs, we see an exponential increase of cases (Fig. 2; Table 3). Containment measures were implemented as a response, which in some countries happened before the first case was confirmed (minus values in Table 3), while some were shortly after the first case, and others occurred later in time. In five countries, which had already reached the peak of the wave before May 31, the length of the critical period (from the start of the wave to its peak) was between 44 days (South Korea and Italy) and 90 days (Sweden), with Germany (61 days) in the middle. All LMICs did not reach the peak until 31 May 2020, because they had particularly long critical periods from 90 to 130 days (Colombia, Mexico, Nigeria, India, and Nepal).

**Table 3:** Critical period of infection and delay to the containment measures in nine target countries

Country	Delay to the first action^a^	Delay to the full action^b^	First case confirmed	Peak date^c^	Critical period^d^	No. of cases at peak	No. of deaths at peak
Germany	−2 days^e^	56 days (except public transport)	27 Jan	27 Mar	61 days	50,871	342
Italy	−7 days^e^	41 days	31 Jan	21 Mar	51 days	53,578	4825
Sweden	39 days	65 days (except public events, public transport, and internal traveling)	31 Jan	24 Apr	85 days	17,567	2152
South Korea	12 days	36 days (except public transport)	20 Jan	03 Mar	44 days	5186	28
Mexico	1 days	32 days	28 Feb	27 May	90 days	78,023	8597
Colombia	−44 days^e^	20 days	06 Mar	Not yet reached	Ongoing	Not yet reached	Not yet reached
India	−4 days^e^	51 days	30 Jan	Not yet reached	Ongoing	Not yet reached	Not yet reached
Nigeria	−36 days^e^	31 days	28 Feb	Not yet reached	Ongoing	Not yet reached	Not yet reached
Nepal	50 days^e^	60 days	25 Jan	Not yet reached	Ongoing	Not yet reached	Not yet reached

^a^Number of days after the first case until the first action

^b^Number of days after the first case until the full action; full action means complete implementation to close schools, close workplaces, cancel public events, ban public gatherings, close public transportations, ban internal traveling, ban international traveling, and promote public campaigns

^c^Date with the peak number of newly confirmed cases; if the event with the highest number of daily cases was on May 30 or thereafter, it was considered “not yet reached” the peak

^d^Number of days after the first case until the peak date with the highest number of cases; if the peak was “not yet reached,” the critical period was considered “ongoing”

^e^Minus values in the delay to the first action mean the countries started with initial containment measures before the first case was confirmed

**Fig. 3:**
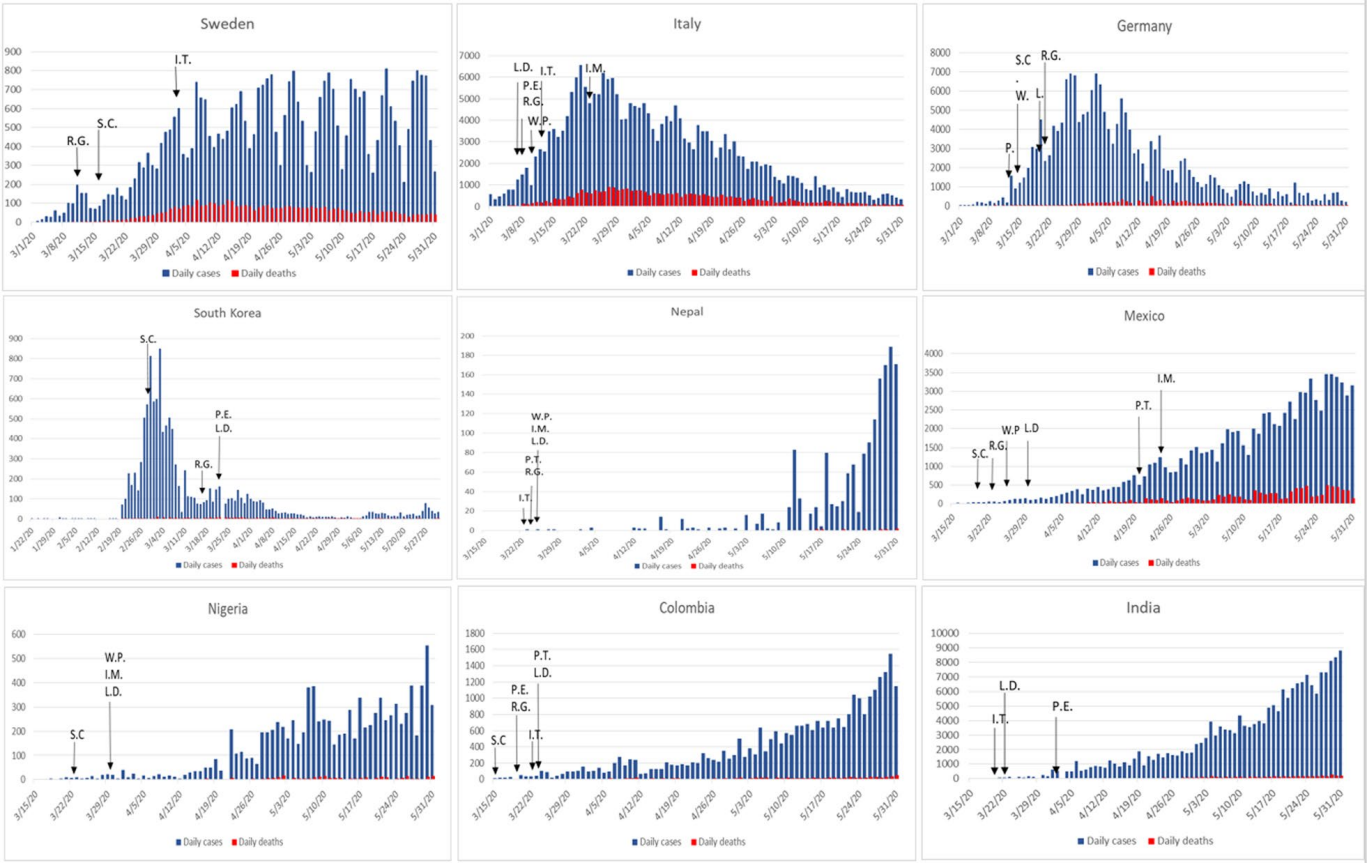
Daily numbers of newly detected COVID-19 infections (“cases”) and deaths. Abbreviations: S.C. = School closure, P.E. = Restriction on public events, W.P. = Workplace closure, P.T. = Closure of public transport, R.G. = Restriction on gatherings, I.T. = Restriction on internal transport, I.M. = Restriction on internal movement, L.D. = Lockdown (partial or complete). Blue = Confirmed cases, Red = Deaths

Figure 3 illustrates that only Germany, Italy, and South Korea show the typical epidemic curve with a sharp increase to the peak and then a slower decrease of cases skewed to the right. The curves of India, Mexico, and Nepal have a similar shape, but only during of the initial part, as they did not reach the peak at the time. Sweden, with a “hands-off” policy and relaxed strategy, has a flat and prolonged curve of new cases; likewise, Nigeria has a flat and prolonged curve, which was limited to the low testing capacity.

As these findings seem to contradict the model predicted by Ferguson et al. to be high and stiff when there is no distancing measure while low and prolonged if containment measures were employed [3], we had a closer look at the stringency of containment measures and peoples` compliance with these measures [16, 18].

#### The stringency of the containment measures and people`s compliance level

**Fig. 4:**
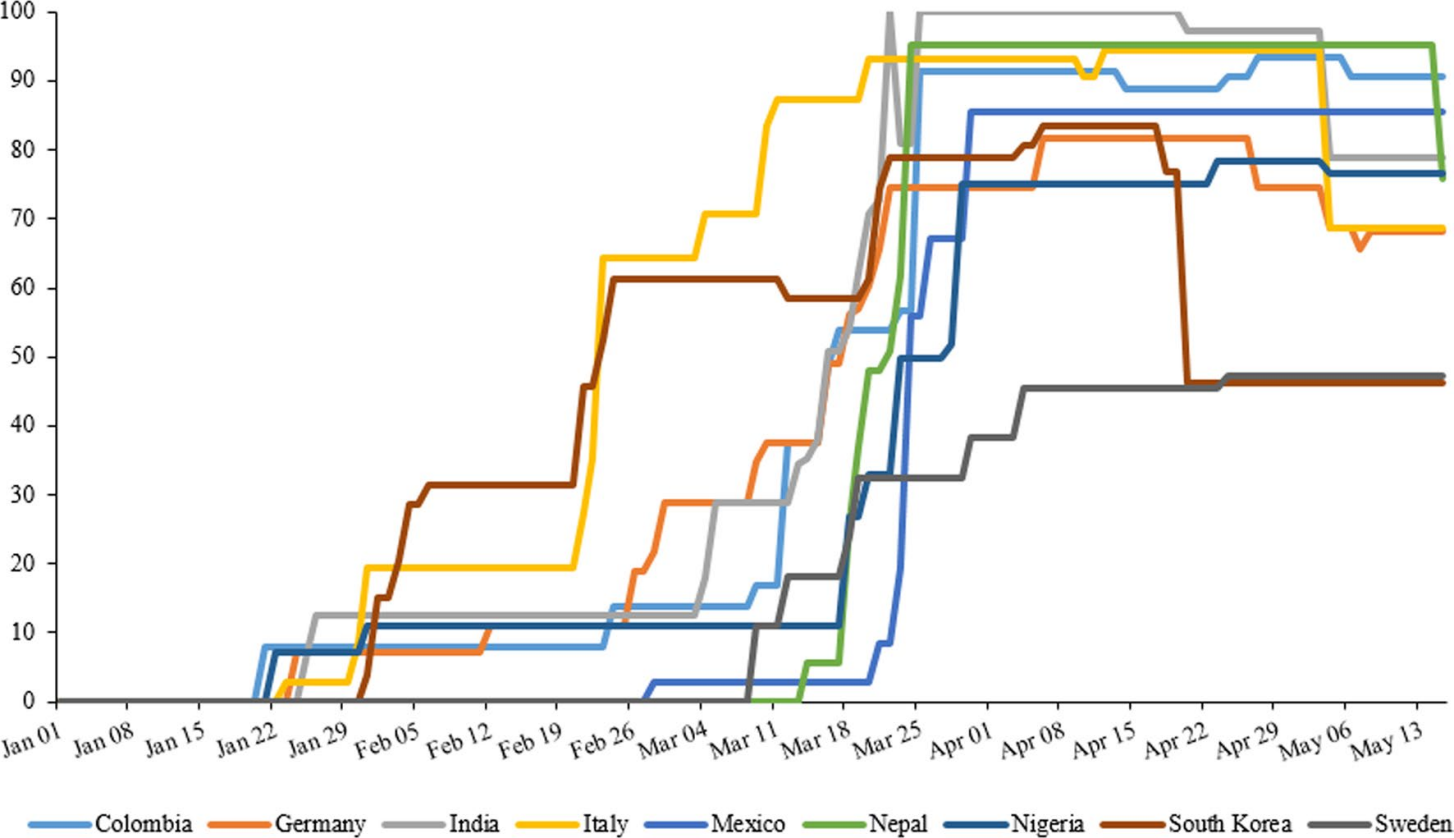
The stringency index reflecting the strength of containment measures in nine selected countries. Methodology for the index estimation followed that of Hale et al., using the information of governmental actions provided by the Oxford COVID-19 Government Response Tracker [7, 8]

Figure 4 and Table 4 shows the SI over time. Some countries started late but were then very fast with containment measures (Nepal, Mexico, and Italy) while others started early but then strengthened the measures step-by-step (Germany, South Korea, and India). Others opted for less stringent measures, particularly Sweden and South Korea (after a month of strict measures). The LMICs were generally more stringent than the HICs.

**Table 4:** Descriptive statistics of stringency index per country and sub-country, for the period of Jan–May 2020

	Mean	SD	Min	Percentiles				Max
				25%	50%	75%	95%	
Countries								
India	58.09	38.87	0	10.19	79.17	96.3	100	100
Nepal	56.86	39.19	0	16.67	81.02	96.3	96.3	96.3
Nigeria	46.08	30.83	11.11	22.22	22.22	80.56	85.65	85.65
Colombia	53.29	36.84	0	8.33	84.26	87.04	90.74	90.74
Mexico	60.11	34.52	0	8.33	82.41	82.41	82.41	82.41
South Korea	51.84	17.94	0	43.52	45.37	56.94	82.41	82.41
Italy	64.71	29.85	0	60.19	69.91	91.67	93.52	93.52
Germany	45.28	27.35	0	11.11	52.78	73.15	73.15	73.15
Sweden	38.98	10.54	0	32.41	43.52	46.3	46.3	46.3
Sub-countries								
India, Kerala	68.09	35.22	10.19	26.85	81.94	100	100	100
Nepal, Kathmandu	69.85	34.88	16.67	22.22	92.59	96.3	96.3	96.3
Nigeria, Abuja	59.51	27.85	22.22	22.22	80.56	82.87	85.65	85.65
Colombia, North Santander	63.79	33.01	8.33	34.26	87.04	87.96	90.74	90.74
Mexico, Nuevo León	60.11	34.52	0	8.33	82.41	82.41	82.41	82.41
South Korea, Daegu	55.47	15.75	31.48	43.52	52.78	75.93	82.41	82.41
Italy, Lombardy	75.63	19.76	19.44	62.96	85.19	91.67	93.52	93.52
Germany, Baden-Württemberg	53.52	22.68	11.11	32.87	64.35	73.15	73.15	73.15
Sweden, Västra Götaland	38.98	10.54	0	32.41	43.52	46.3	46.3	46.3

**Fig. 5:**
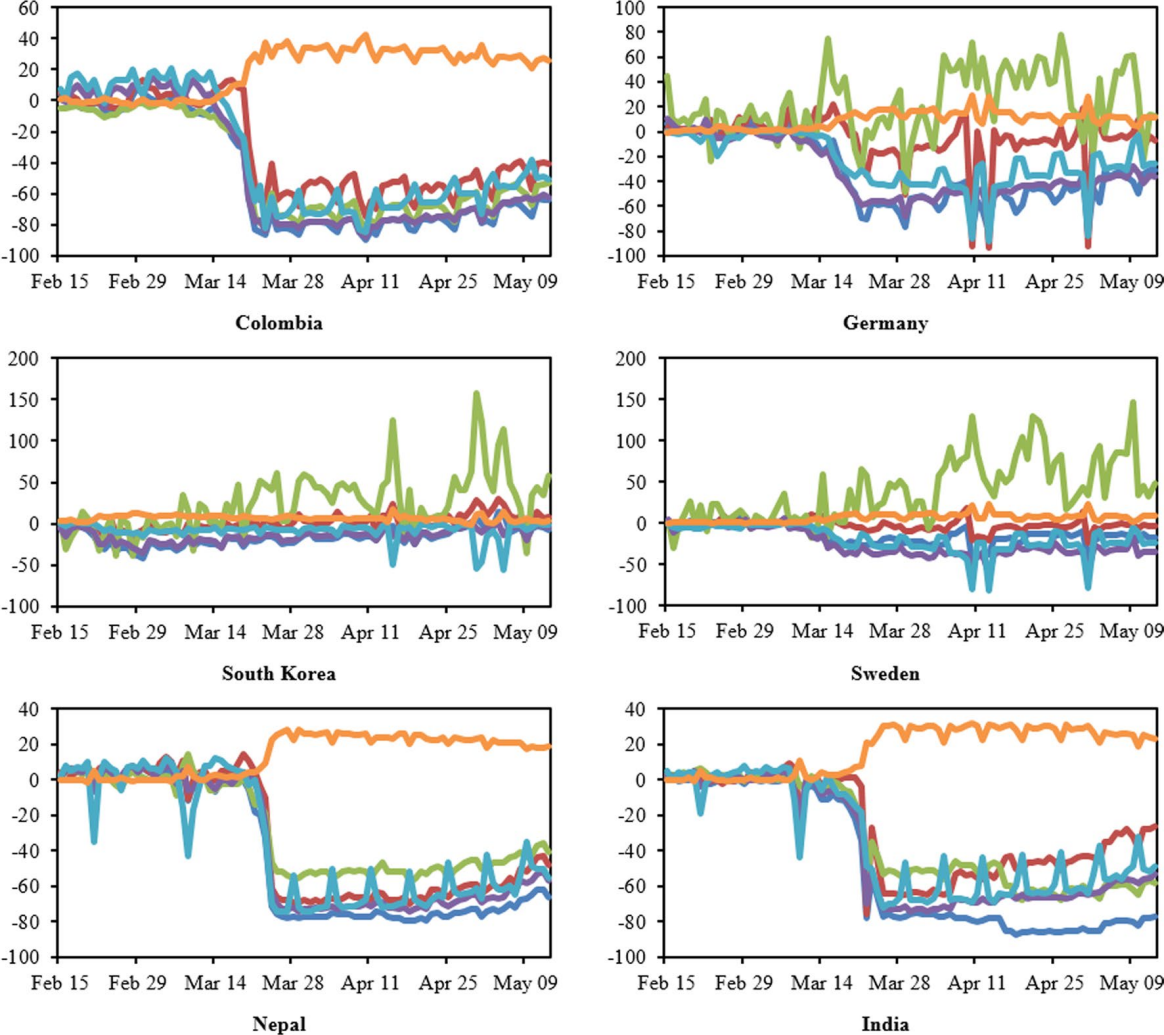
Human mobility after the start of containment measures in six countries (Colombia, Germany, South Korea, Sweden, Nepal, and India). Blue = Retail and recreation, Red = Grocery and pharmacy, Green = Parks, Purple = Transit stations, Light blue = Workplaces, Orange = Residential

Human movement after the introduction of containment measures in six countries are given in Fig. 5, which shows that in Colombia (similar to Italy, India, Nigeria, and Nepal), people stayed at home and did not follow many extra-domestic activities. Germany (similar to Mexico) illustrates a less strict restriction of mobility: people stayed more at home with limited recreational activities strictly, but used the public transport and visited public parks more frequently. In Sweden and South Korea, the less stringent containment policy led people to continue going to work, using pharmacies/groceries, and using public transports almost as usual, and increasing visits to parks rather than other recreational activities.

#### Containment measures shaping the epidemic curve (Interrupted time-series analysis)

**Fig. 6:**
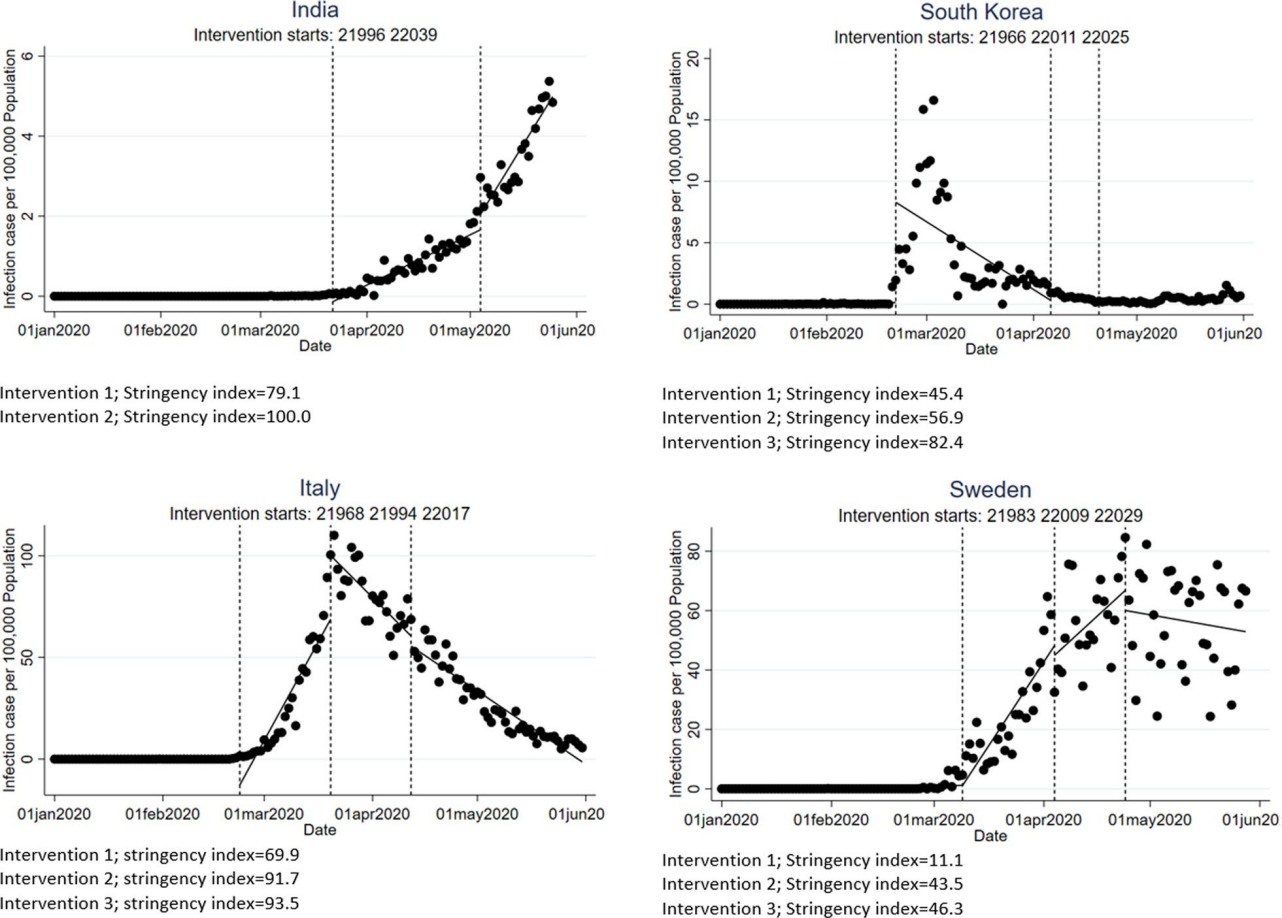
Infection rates by the stringency index in India, South Korea, Italy, and Sweden

Findings from the time-series analysis for the infection and death rates showed different patterns across the nine countries (Tables 5 and 6; Fig. 6).

- India: At baseline (prior to any intervention), infection rates showed a slight increase, which reversed temporarily at the time of the first intervention (−0.148; p < 0.05) before starting to increase again at the following intervention (0.14; p < 0.05). At sub-country level in Kerala, a fairly similar scenario was observed with exception for the rate at and after the first intervention. The death rate followed the scenario of the infection but at a slower pace.
- Nepal: The baseline trend did not indicate a statistically significant rate of change, but showed an increasing trend after the first intervention (0.004; p < 0.05). Although a slightly different rate of change was observed at sub-country level (Kathmandu), generally, the rate of reduction followed a similar trend of increasing infection rates throughout the period. Mortality rates were low and therefore no pattern of change was observed.
- Nigeria: The baseline trend did not indicate a rate of change but showed an increasing trend throughout the follow-up period. In Abuja, the same trend was observed but at a higher rate; however, a decreasing trend in infection rate was observed after the second intervention. The death rates at country and sub-country levels followed the same trend but with an increasing death rate at baseline and after intervention one but decreased following to the second intervention. Nevertheless, none of these changes showed a significant trend.
- Colombia: The increasing infection rates at baseline were minimal but significant and continued to increase significantly in spite of the second and third intervention. In North Santander, the rate of infection started with an increasing trend but responded to both interventions with a mild decline. The same scenario was observed for the death rates at country and sub-country levels.
- Mexico: Mexico had minimal changes in the stringency index after the first intervention but was successful in reducing the infection and death rates significantly thereafter (infection: −2.676; p < 0.05, death: −0.359; p < 0.05). However, after this initial success both infection and death rates increased shortly after the intervention; this trend was similar in Nuevo Leon.
- South Korea: South Korea had a continuous increase in the SI with three different intervention episodes. At baseline, the country showed an increasing infection rate until the first intervention before cases started to decline significantly (−0.181; p < 0.05), which was continued after the third intervention (−0.119; p < 0.05). Daegu Province (where more than half of the cases occurred) followed a similar trend, but the increasing stringency measures succeeded in reducing the overall infections (−0.036; p < 0.05). The impact of the interventions on death rates was even more significant both at country and sub-country level.
- Italy: The rate of infection was much higher in Lombardy compared to the whole country. Nevertheless, containment measures managed to reduce infection rates significantly at both levels (country: −1.163; p < 0.05; Lombardy: −1.520; p < 0.05). The same results were obtained for the mortality, which equally maintained a significant decline after the interventions (country: −0.174; p < 0.05; Lombardy: −0.325; p < 0.05).
- Germany: Germany showed an increasing incidence before imposing containment measures, but this rate started to significantly decline after the first intervention; this decline continued after the second and third interventions so that the rates could be reduced by −0.232 (p < 0.05). Baden-Württemberg followed a fairly similar trend. The death rates at country- and sub-country level showed similar trends.
- Sweden: Sweden started late in imposing containment measures with a low SI. Albeit the minimal average of the SI, the country managed to reduce the rate of infection immediately after the first intervention and continued through the following two interventions, although the reduction was statistically insignificant (−0.21: p > 0.05). The picture was different in Västra Götaland where the containment measures failed to generally reduce the rate of infection significantly. The death rate followed a similar trend for both country and sub-country level.

**Table 5:** Interrupted time-series regression of infection rate per 100,000 in relation to countries- and sub-countries stringency Indices (Intervention)

	Cases per 100,000								
**Country level**	India	Nepal	Nigeria	Colombia	Mexico	South Korea	Italy	Germany	Sweden
Baseline trend	0.0001*	−0.001	7.9 × 10^–6^	0.001*	0.006*	0.004	0.002	0.136*	0.025*
At intervention 1	−0.148*	−0.01	0.003	0.561*	−2.676*	8.160*	−12.819*	61.896*	−0.073^a^
Trend after intervention 1	0.042*	0.004*	0.006*	0.114*	0.461*	−0.181*	3.135*	−1.368*	1.787*
At intervention 2	–	–	–	–	–	0.626	31.33*	–	−3.262
Trend after intervention 2	–	–	–	–	–	0.124*	−4.857*	–	−0.718
At intervention 3	0.384	−0.509	0.604*	0.946	–	−0.119	−4.98	−1.213	−6.781
Trend after intervention 3	0.098*	0.142*	0.02*	0.316*	–	−0.069*	0.557	0.973*	−1.303
General trend after all levels of intervention	0.14*	0.146*	0.025*	0.432*	0.467*	0.016*	−1.163*	−0.232*	−0.21
**Sub-country level**	Kerala	Kathmandu	Abuja	North Santander	Nuevo León	Daegu	Lombardy	Baden-Württemberg	Västra Götaland
Baseline trend	0.005*	0.006	0.014*	0.022*	0.021*	6.33*	3.33*	0.838*	0.401*
At intervention 1	0.33*	−0.117	0.091	0.432	−1.214*	105.136*	130.759*	75.703*	12.026^a^
Trend after intervention 1	−0.011*	−0.006	0.076	−0.021	0.164*	−10.589*	−5.785*	−2.468*	0.433
At intervention 2	–	–	–	–	–	–	–	–	–
Trend after intervention 2	–	–	–	–	–	–	–	–	–
At intervention 3	0.395*	0.127	0.91	0.439	–	30.13	−3.432	7.368	14.163
Trend after intervention 3	0.170*	0.026	−0.069	−0.02	–	4.223*	0.934	1.646*	−0.639
General trend after all levels of intervention	0.165*	0.026	0.021	−0.019	0.185*	−0.036*	−1.52*	0.016	0.195

Intervention 1 measured at 50th, intervention 2 measured at 75th and intervention 3 measured at 95th percentile

^*^Significant change (p < 0.05)

^a^intervention 1 is measured at 10th percentile instead of 50th (only in Sweden)

**Table 6:** Interrupted time-series regression of death rate per 100,000 in relation to countries- and sub-countries stringency Indices (Intervention)

	Deaths per 100,000								
**Country level**	India	Nepal	Nigeria	Colombia	Mexico	South Korea	Italy	Germany	Sweden
Baseline trend	2.3 × 10^–6^	0.000	0.001*	0.002*	0.000*	0.001	0.001	0.0003*	0.0002
At intervention 1	−0.004*	–	–	–	−0.359*	0.039*	−1.562*	1.056*	−1.033*
Trend after intervention 1	0.001*	–	–	–	0.055*	0.002*	0.301*	0.034*	0.303*
At intervention 2	–	–	0.022*	–	–	−0.015	6.704*	–	0.054
Trend after intervention 2	–	–	0.001	–	–	−0.007*	−0.413*	–	0.073
At intervention 3	0.027*	0.002	−0.012	0.101*	–	−0.006	−0.144*	−1.348*	−4.665
Trend after intervention 3	−0.000	0.001	−0.001	0.008*	–	0.005*	−0.062	−0.778*	−0.451*
General trend after all levels of intervention	0.001	0.002	0.0002	0.01*	0.055*	−0.0004*	−0.174*	−0.043*	−0.074
**Sub-country level**	Kerala	Kathmandu^a^	Abuja	North Santander	Nuevo León	Daegu	Lombardy	Baden–Württemberg	Västra Götaland
Baseline trend	7.78 × 10^–6^	–	0.001	0.003*	0.002*	0.027	0.335*	0.003*	0.018*
At intervention 1	–	–	0.006	–	–0.013	0.696*	28.27*	2.596*	0.146
Trend after intervention 1	–	–	−0.001	–	0.015*	−0.009	−0.679*	−0.01	0.271
At intervention 2	–	–	–	–	–	–	–	–	−0.949
Trend after intervention 2	–	–	–	–	–	–	–	–	−0.248
At intervention 3	−0.001	–	−0.037	−0.035	–	−0.745*	−4.803	−1.633*	–
Trend after intervention 3	0.002	–	0.009	−0.005	–	−0.036*	0.019	0.026	–
General trend after all levels of intervention	0.002	–	0.008	−0.002	0.017*	−0.018*	−0.325*	0.02	0.041

Intervention 1 measured at 50th, intervention 2 measured at 75th and intervention 3 measured at 95th percentile

^*^significant change (p < 0.001)

^a^No reported cases

^b^Intervention 1 is measured at 10th percentile instead of 50th percentile (only in Sweden)

## Discussion

### Effective containment measures: scoping review

Main messages from the scoping review about the effect of containment measures on infection rates were that containment measures, particularly when initiated early, could reduce the progression of the disease transmission (Italy) [21], reduce the basic reproduction number R_0_ from 2.3 to 0.15 (India) [36], and delay the peak of the epidemic by 3 days in modelling studies [29, 35]. A combination of containment measures is more effective, leading to a reduction of secondary cases by 90 to 99% in South Korea [25]. In Spain and Italy, particularly the second intervention, when added to the first one, led to the reduction of infections [34]. However, the lockdown of mild and asymptomatic cases alone did not have much effect in modelling studies [32], and prolonged lockdown beyond 42 days did not have an additional benefit (India) [31]. Furthermore, fever checking in airports needs a very high coverage to be effective, particularly considering all asymptomatic arrivals [35]. Finding from our review came to the same conclusion—that containment measures ware effective in reducing the transmission dynamics, but only in HICs and only minimal in LMICs.

### Wealth and disease burden

Our participant countries represented a range of income groups whereby Germany, Sweden, South Korea, and Italy belong to the high-income category in contrast to the LMIC group, which engulfs the rest of the countries. The country’s wealth status has apparent implications, which is reflected in the distribution of the cumulative infection and death rates (Fig. 2). Higher income nations displayed a sharp increase of both infection and death rates at the early stages of the pandemic, which slowed down 4 to 6 weeks later, with the exception of Sweden which had a less-pronounced decline due to the “relaxed” containment policy. Among the LMICs, the increase of infections was stiffer and started earlier in time and continued throughout without reaching the peak during the observation period. This difference warrants further investigation.

### Assessing the containment measures in LMICs and HICs

The SI as a summary measure of the different components of the containment strategies implemented over time revealed the following (Fig. 4). High and long lasting SIs were found particularly in LMICs (India, Nepal, and Colombia) and lower SIs and/or shorter duration were observed in HICs, particularly in Sweden, South Korea, and Germany. Mobility data (Fig. 3) reflecting the compliance of people with the lockdown measures also seem to show that populations in LMICs were more compliant than those in HICs.

In South Korea, Germany, and Italy, the containment measures were successful in reducing infection rates and deaths, particularly when several restrictions were combined. In Sweden, the effect was also present but not statistically significant, which is explained by the minimal changes of lifestyle during the epidemic. Conversely, the effect in LMICs was disappointing in relation to strict containment measures imposed, particularly in India, Nepal, and Colombia. Despite the fact that peoples’ compliance with the measures appears satisfactory, these countries have achieved minimal and only temporary reduction of infection rates or deaths. Also at the sub-country level, no apparent or sustained success could be observed. For instance, in Kerala, India, containment measures were particularly strict but only a minimal transient effect on transmission reduction could be observed.

### Likely causes for the unsuccessful containment measures in LMICs

Data from LMICs are usually less reliable than those from HICs. Systematic testing is rarely being done, not even in symptomatic cases, and the number of infections and deaths is mainly health service based. However, the information on the sharp increase of new infections and deaths is worrying enough although the real burden is most likely to be much higher.

We assume that the high proportion of people working in the informal economic sector, particularly those in vulnerable employments (India, 74.5% vs. Germany, 5.7%), explains why lockdown measures are impossible to comply with in poverty areas. “No work, no food” illustrates that the majority of people in these areas cannot afford to stay at home. This is not captured by our human mobility analysis which rests on the ownership of a smart phone and does not reflect the movements of the poor. In other words: the lockdown measures are not followed by a large population segment which drives the virus transmission.

### Comparison with similar studies from across the world

The additional literature search after completing the scoping review and the analysis of data at the earlier stages of the pandemic added the following information: A study including 41 countries showed that containment measures including non-pharmaceutical interventions were effective in reducing Covid-19 transmission, with some measures greater than others [39]. A recent study from India found that the time-varying reproduction number (*R* (t)) was reduced in several states as a result of various containment measures [40]. It was shown that the reproduction number increases with higher population density as it facilitates the transmission of the virus. Thus, mobility restrictions could markedly bring down the COVID-19 spread in densely populated regions (see also Table 1 on population density) [41, 42]. However, such restrictions could not be implemented for a long period in LMICs, where the proportion of people belonging to the informal sector is high, as discussed above.

Other studies showed the impact of containment on mobility. A study by Barbieri et al. found in ten countries a decrease of private mobility during the first wave of the pandemic [43]: people abstained from walking (11.3% reduction in Iran), cycling or driving a car (10.2% and 13.7% reduction, respectively in Ghana). Also, the use of public transportation decreased the most significantly in Iran (18.7% less use of trams) and Australia (7.2% less use of trains) and Norway (decreased use of buses, −19.4%, and airplanes, −4.9%).

Another recent study which summarized guidance for low-income countries said that authorities in African countries could learn from China to improve emergency responses to pandemics, be more proactive, and be committed to planning and performing long-term plans for coming pandemics. Furthermore, there should be a promotion of hygiene and public participation as a routine application in all communities in Africa. Liaising with medically sophisticated countries will facilitate real-time information, assuring that gaps between advanced countries and LMIC like Africa are reduced. African countries should also increase their capacities to make their anti-epidemic elements such as personal protective equipment and testing kits to flatten the curve [44].

These study results were in line with the results of the here presented study (reduced mobility during the lockdown, population density as a risk factor for COVID-19 spread), but did not distinguish between rich and poor countries.

## Study limitations

This study has utilized existing records of national and sub-national infection and death cases as reported by established sources [7–14], including the pragmatic measure of SI and Google mobility data as described elsewhere [16, 18]. Just like the case of data reporting for other diseases, the authors acknowledge the possible incorrect disease classification of diagnosis particularly in LMICs including the frequent underreporting of mortality and morbidity data. However, information sources in the current study have been most useful in several recent epidemiological studies. In addition, the SI is deemed as a novel index to be applied in research, but our results suggest plausible interpretations of this index which appear to follow what was expected in the corresponding countries. Since the Google mobile data is prone to several limitations as acknowledged by their providers [18], we attempted to interpret this information more cautiously and only integrated their records in the descriptive statistics to aid the study discussion.

## Conclusion

Compared to HICs, the transmission dynamics seem to follow different paths in LMICs requiring different and more context-specific strategies in order to contain the spread of the virus and protect the most disadvantaged societies. This is certainly a novel challenge for the global health community and experiences from local settings will likely help to shape national and global policies and find new ways of dealing with the COVID-19 pandemic and its disastrous impact on peoples` lives.

## Supplementary Information

**Additional file 1: Table S1**. Description of the included studies in literature review.

## Data Availability

Data openly available in a public repository that does not issue DOIs. The data that support the findings of this study are openly available in the Humanitarian Data Exchange at https://data.humdata.org/dataset/novel-coronavirus-2019-ncov-cases; The Humanitarian Data Exchange: Government of Nepal at https://covid19.ndrrma.gov.np/; Gobierno de México. Datos Abiertos—Dirección General de Epidemiología at: https://www.gob.mx/salud/documentos/datos-abiertos-152127; Nigeria Centre for Disease Control at: https://covid19.ncdc.gov.ng/report/#!; Instituto Nacional de Salud at https://infogram.com/1pyg0lgpndvwweh3yx1exq1dq1uy6l7xmpg; Daegu Metropolitan City at: http://covid19.daegu.go.kr/; Variation in Government Responses to COVID-19 at: https://www.bsg.ox.ac.uk/research/research-projects/covid-19-government-response-tracker. All accessed September 15, 2020.

## Abbreviations

COVID-19: Corona virus disease, 2019
GDP: Gross domestic product
HIC: High-income countries
ICU: Intensive care unit
LMIC: Low-middle income countries
MIC: Middle-income countries
NPI: Non-pharmaceutical interventions
SI: Stringency index
