# Supplementary material for: Factors shaping the COVID-19 epidemic curve: a multi-country analysis
Source: BMC Infect Dis. 2021 Oct 2;21:1032. doi: 10.1186/s12879-021-06714-3 (PMC8487341; doi:10.1186/s12879-021-06714-3)
Supplement: Supplementary file 1 — Additional file 1: Table S1. Description of the included studies in literature review. [file 12879_2021_6714_MOESM1_ESM.docx]

**Additional file 1**

**Table S1. Description of the included studies in literature review.**

| **No.** | **Authors (year)** | **Title** | **Country** | **Type of study** | **Status of the publication** | **Analysed**  **measure** | **Methodology** | **Instruments** | **Results** |
| --- | --- | --- | --- | --- | --- | --- | --- | --- | --- |
| 1 | Sebastiani et al, (2020) | Covid-19 epidemic in Italy: evolution, projections and impact of government measures. | Italy | Modelling study | Published | Governmental measures | secondary data analysis Data on accumulative cases of Covid-19 from 1 to 31 of March 2020 in 107 provinces | the time sequence of the cumulative number of cases (*Covid-19 CI growth rate*) the study used comportamental model which considered asymptomatic cases | The time lag between the start of the implementation of the restriction measures and the measurable reduction of the Covid-19 CI growth rate was approximately 7–10 days. On March 31 the epidemic growth rate reached a peak in 87 out of 107 Italian provinces, on average 13.6 days after the measures were implemented. The comparison of the curves of the epidemic in different Italian regions in relation to the time when the government measures were introduced (9th to 12th of March 2020), suggests that the earlier the measures were taken in relation to the phase of the epidemic in that particular region, the lower the cumulative incidence achieved during this epidemic wave. |
| 2 | Tobias, (2020) | Evaluation of the lockdowns for the SARS-CoV-2 epidemic in Italy and Spain after one month follow up. | Italy -Spain | Interrupted time-series design | Published | lockdown | Comparative analysis of data  Data on daily cases were collected between February 24th and April 5^th^ | incident cases, deaths, and intensive care unit admissions (ICU) in both countries before and after their lockdowns | Before the lockdown, the daily percent increase of all the incidence outcomes was higher in Spain (38.5% for diagnosed cases, 59.3% for deaths, and 26.5% for ICU admissions) than in Italy (21.6%, 32.8%, and 16.7%, respectively). During the first lockdown period, both countries show similar daily trends (12.5%, 13.7%, and 3.7% in Italy; and 11.9%, 17.6%, and 9.6% in Spain). Thus, during the first lockdown the daily increase in incident data was considerably reduced. During the second and more restrictive lockdown. Specifically, in Italy all outcomes start declining; diagnosed cases if −2.0%, daily deaths of −0.2%, and ICU admissions of −16.8%. In Spain also declined −2.7%, −1.8%, and ICU −5.6%, respectively. Efforts overtaken are being successful in flattening the epidemic curve |
| 3 | Oksanen et al, (2020) | Regulation and Trust: 3-Month Follow-up Study on COVID-19 Mortality in 25 European Countries. | Multiple countries, including GermanySweden , Italy | Cross sectional | Published | Governmental measures | Comparative analysis of data  Data form January 22 to April 14, 2020 in 25 European countries | Multilevel mixed effects linear regression models focused on 84 days of the COVID-19 epidemic and daily mortality. Comparison placed before or after the first COVID-19 death (days). | The most impacted countries in Europe by April 14 are Italy, Spain, and France. These countries were also significantly late to implement national restrictions. For example, Italy placed national restrictions almost 2 weeks after the first COVID-19 incident (13 days). In Scandinavia, for example, Sweden has adopted less restrictions than Denmark, Finland, and Norway. Sweden also had a higher number of deaths per inhabitants at the time of this article. Major differences between the 25 countries were found in reaction times to the crisis. Late reaction to the crisis increased the deaths. |
| 4 | Bruinen de Bruin et al, (2020) | Initial impacts of global risk mitigation measures taken during the combatting of the COVID-19 pandemic. | Multiple countries, including Italy, Germany and South Korea | Review | Published | Governmental measures | Narrative analysis | Review of literature on measures implemented by countries around the world facing the current COVID-19 outbreak | Mobility restrictions, physical distancing, hygienic measures, socio-economic restrictions, communication and international support mechanisms were identified in the study. For instance, in socio-economic restrictions, Google showed that visits to shops, parks or railway stations fell steeply in most European countries between February 16 and March 29. Reuters reported that in Italy, retail and recreational trips were down by 94%. For physical distancing, European governments increasingly applied a combination of communication strategies and enforcement strategies, Germany established on March a fine of up to 25000 EUR. For Hygiene Measures, in the majority of EU, the public should not wear face masks and in parts of Asia (including South Korea), all citizens have been encouraged to wear face masks in public. Italy has joined a small but growing number of European countries making the wearing of face masks outside the home compulsory. |
| 5 | Signorelli et al, (2020) | COVID-19 in Italy: impact of containment measures and prevalence estimates of infection in the general population. | Italy | Cross sectional | Published | Governmental measures | Narrative analysis | Number of deaths, period prevalence and observations of epidemic curve | The initial measures to suspend flights from China and air- ports’ checkpoints with thermoscan did not seem to have a significant effect in containing the epidemic. The implementation of a “red zone” in Lombardy effectively contained the spread of the infection within that area. On the other hand, the “red zone” measure did not have the same effect outside that area. In fact, three of the neighbouring provinces (Bergamo, Brescia, and Piacenza) recorded the highest incidence rates in the weeks following the establishment of the red zones. The lockdown implemented as of 9 March had a positive impact in the central and southern regions of Italy, while other actions appeared to be less effective. Overall, containment measures “red zone” and mitigation (general lockdown) can be effective if taken at an early stage of the epidemic. |
| 6 | Nussbaumer-Streit et al, (2020) | Quarantine alone or in combination with other public health measures to control COVID-19: a rapid review. | Multiple countries, including South korea | Review | Published | Quarantine (alone or in combination with other public health measures) | narrative analysis with tabular form | A systematic review form several databases | Review presented one study from South Korea which was conducted by Choi (2020). The paper simulated the outbreak for South Korea and estimated that there would be nearly 5 million COVID‐19 cases without any measures. By implementing prevention and control measures that are able to reduce the transmission rate by 90% or 99% the number of COVID‐19 cases would be only a fraction, at 0.5% or 0.4%, respectively. In general, this review evidenced that quarantine to be important in reducing the number of people infected and the number of deaths. Results showed that quarantine was most effective, and cost less, when it was started earlier. Combining quarantine with other prevention and control measures had a greater effect than quarantine alone. |
| 7 | Sjödin et al, (2020) | Only strict quarantine measures can curb the coronavirus disease (COVID-19) outbreak in Italy, 2020. | Italy | Modelling study | Published | Quarantine in different scenarios | Secondary data analysis | Estimation of the number of total secondary cases during the 14-days period, time spent out of house and number of person in quarantine. Standar reproductive number=2.1 | The model predicted that for an average household size of two persons with complete, near-complete, medium and no community quarantine, 3, 4, 7 and 11 secondary cases are estimated during the lockdown. With an average three-person household size, 7, 8, 12 and 20 secondary cases are estimated, respectively. With a six-person average household size, 16, 19, 29 and 43 secondary cases would be predicted to occur over the 14-days period, respectively. Researchers suggest that the degree of quarantine adherence needs to be very high regardless of population size in order to be effective. A less strict community quarantine could still flatten the curve of the outbreak compared to no quarantine. In any case, quarantine adherence is key for reducing the outbreak, but some transmission will still occur within households, therefore more efforts at house level are needed. |
| 8 | Kim et al, (2020) | Prediction of COVID-19 transmission dynamics using a mathematical model considering behavior changes in Korea. | Korea | Modelling study | Published | Behaviour changes (behaviour changes in transmission dynamics related to the implementation of the strategies) | Secondary data analysis  Descriptive analysis | Mathematical model of COVID-19 transmission based on the SEIR model with a hospital-quarantined group.  Modified version of the model of prevalence-based behavioral change proposed by Perra et al. (global, prevalence based spread of the fear of the disease) for the behavioral changes of the susceptible group. | The model indicated that the behavior-changed group reduction rate in transmission influences the model results significantly. The rate of individuals moving from the susceptible to the behavior-changed susceptible group is 0.02. Using these estimated parameters, the model predicts that circa 13,800 cases will occur nationwide and 11,400 cases in the Daegu/Gyeongbuk zone by the middle of June. In the model, a decline in the transmission rate of behavior-changed susceptible individuals, results in a decrease in the total number of confirmed cases and the duration of the outbreak. The researchers assumed that the susceptible persons that implemented behavioral changes maintain a low transmission rate. However, an increment in the social interaction and a desensitization of the behavior-changed group with a decreasing number of patients is expected after schools reopening on March 24. This situation may generate another cluster outbreak stemming from unconfirmed positive cases. |
| 9 | Goldstein et al, (2020) | Temporal rise in the proportion of younger adults and older adolescents among coronavirus disease (COVID-19) cases following the introduction of physical distancing measures, Germany, March to April 2020 | Germany | Cross-sectional | Published | Role of age groups at propagating the infection (before and after implementation of the measures) | Secondary data analysis | - Relative risk (RR): to evaluate temporal increase in the share of a given age group among all cases of infection.  - Fisher’s exact test to estimates for pairwise OR. | People between 15–34 years old (particularly the individuals from 20–24 years) contribute in the increment of the incidence of SARS-CoV-2 with time compared with theage group 35–49 years old and children aged 10–14 years.  RR for different age groups for the later period (weeks 13–14) vs the early period (weeks 10–11): the highest RR was between aged 20–24 years 1.4 (1.27-1.55 95% CI), followed by individuals aged 15–19 1.14 (0.99-1.32 95% CI), 30–34 1.07 (0.99-1.16 95% CI) and 25–29 years 1.06 (0.98-1.15 95% CI) . For the age group 20–24 years, the OR relative to any other age group for being a case during the later vs the early period was significantly above 1 (age group/OR/(95%C)I: 25-29/1.32/(1.15-1.53), 30-34/1.31/(1.14-1.51), 35-39/1.48/(1.29-1.71), 40-44/1.55/(1.35-1.79), 45-49/1.7/(1.48-1.94). |
| 10 | Gregori et al, (2020) | A first estimation of the impact of public health actions  against COVID-19 in Veneto (Italy) | Italy | Modelling study | Published | Policies implemented in the Veneto region (Italy) to contain the COVID-19 epidemic outbreak (effect of the policies implementation in the hospitalisation curves) | Secondary data analysis  Descriptive analysis | - A Bayesian changepoint detection method (BCPDM) based on the procedure proposed by Barry and Hartigan. Then, a piecewise polynomial model was considered to fit the data in the first period before the detected change point. | At comparing the hospitalisation curves observed and estimated there was a downtrend in the total of COVID-19 hospitalisations after the introduction of the containment strategies. 78 hospitalizations per day as of 27 March (95% CI 75.09 to 80.94) was estimated as the slowdown effect of the epidemic growth. A specific changepoint in the hospitalisation growth was seen on the 12th of March, 17 days after the first decree law that established a lockdown in some towns in the Veneto region. |
| 11 | Ambikapathy et al, (2020) | Mathematical Modelling to Assess the Impact of Lockdown on COVID-19 Transmission in India: Model Development and Validation | India | Modelling study | Published | Lockdown scenarios: 4, 14, 21, 42, and 60 days (impact of the scenarios on COVID-19 transmission) | Secondary data analysis  Descriptive analysis | A dynamic mathematical model for prediction of the future infected population with COVID-19 | A 4 days scenario showed no significant change in the predicted infected cases compared to a scenario without intervention. The 21, 42 and 60 days preventive scenarios demonstrated a significant reduction in the number of infected cases. For the 21-day lockdown, the number of estimated cases decreased from 378,036 (non-intervention) to 70,424 in 110 days. In addition, 42,950 were further reduced for the 42-day lockdown. However, there was no significant change in the predicted number of infections between the 42-day and 60-day lockdowns.  Regarding the assessment of the situations of enhanced exposure due to the mass gathering in transit stations and shopping malls on the day before the lockdown: It was observed that even for 2-fold augmentation in transmission (r=2), the predicted number of infected people incremented to 450,618 despite the 21-day lockdown. |
| 12 | Mandal et al, (2020) | Prudent public health intervention strategies to control the coronavirus disease 2019 transmission in India: A mathematical model-based approach | India | Modelling study | Published | - Airport screening.  - Quarantine (mitigation of COVID-19 impact). | Secondary data analysis  Descriptive analysis | Mathematical models of infectious disease transmission (Susceptible-Exposed-Infectious-Recovered (SEIR) | - Airport screening of symptomatic arrivals will lead to a delay of 2.9 days in the predicted “average time to epidemic” (days to reach a prevalence of 1000 cases in India). In order to get a delay of 20 days on this parameter an additional 90% coverage in the screening of asymptomatic passengers will be needed (in an scenario where R_0_ is 2 and the relative infectiousness, asymptomatic versus symptomatic individuals is 0.1).  - In an optimistic scenario (where the basic reproduction number R_0_ was 1.5 and there was no infectiousness of asymptomatic cases) could decrease the cumulative incidence by 62% and the peak prevalence by 89%. In contrast in a pessimistic hypothetical scenario (where R_0_ was 4 and the infectiousness of asymptomatic cases was the half in relation of the infectiousness of symptomatic cases) the estimated impact in the cumulative incidence will drop to 2% and 8% for the peak prevalence. The predictions are under the assumption where 50% of symptomatic cases are in quarantine within three days of developing symptoms. |
| 13 | Manchein et al, (2020) | Strong correlations between power-law growth  of COVID-19 in four continents and the  inefficiency of soft quarantine strategies | Multiple countries, including Italy | Modelling study | Published | Social isolation  Social interaction | Secondary data analysis  Descriptive analysis | - Power-law growths.  - Distance correlation (DC).  - Variation of the SEIR (Susceptible–Exposed–Infectious–Recovered) epidemic model; that contains 6 Ordinary Differential Equations (ODEs) | Strategies were applied in Italy and France on the 28th of March. The social isolation intervention (“identification and isolation of asymptomatic and mild symptomatic cases”) was not sufficient to significantly reduce the total number of confirmed infected individuals for France, since the last value θ^eff^ = 1.90 indicates a large level of social interaction in the country. On the other hand, for Italy, a considerable reduction was seen. Large social distances (θ = 0.8) are the most efficient scenarios to induce an accentuated reduction of the growth and a fast convergence to the maximal number of confirmed cases. The combination between social interaction and the high degree of isolation of infected individuals could be implemented to prevent economic disasters due to the impossibility of working in the population. |
| 14 | Tomar et al, (2020) | Prediction for the spread of COVID-19 in India and effectiveness of preventive measures | India | Cross-sectional | Pre-proof | Social isolation and lockdown | Secondary data analysis  Descriptive analysis | Transmission rate analysis (r) | Preventing strategies such as social isolation and lockdown have an effect on the reduction of the spread of the virus and subsequently they make the curve flat. Social isolation” is the complete lack of contact between an individual and society, while lockdown is an emergency protocol that usually prevents people from leaving an area”. Transmission rate analysis (r) scenario: value of r before lockdown= 2.3 (i.e. an infected individual can infect the 2.3 persons) and after lockdown it is reduced to 0.15. |
| 15 | Korea Centers for Disease Control and Prevention, (2020) | Coronavirus Disease-19: The First 7,755 Cases in the Republic of Korea | Korea | Cross-sectional | Published | Demographic and epidemiological measures | Secondary data analysis.  Descriptive analysis | Demographics and case fatality proportion.  Age distribution and sex ratio among Daegu, Gyeongbuk, and others regions. | Case fatality proportion was the highest among people aged ≥ 80 years in Daegu, followed by those aged 70–79 years in Daegu, and elderly persons ≥ 80 years old in Gyeongbuk. The outbreak generally began with the younger age group, followed by the elderly population. There were 66 laboratory-confirmed fatal cases of COVID-19, as of March 12th, 2020. The median age was 77 years (range, 35–93 years), and female-to-male ratio of 44:56. Of 63 cases, 96.8% were reported to have comorbidities: 47.6% hypertension, 36.5% diabetes, 16% neurodegenerative disorders, and 17.5% pulmonary diseases. There were 71.2% of fatal cases in Daegu and 24.2% were from Gyeongbuk. There were 5 patients who succumbed to death at home, and COVID-19 was identified as the cause of death in 11 individuals after their death. The median interval between onset of symptoms and death was 10 days (range 1–24 days), while the median interval between date of hospitalization and date of death was 5 days (range 0–16 days). |
| 16 | Shim et al, (2020) | Transmission potential and severity of COVID-19 in South Korea | Korea | Cross-sectional | Published | By using the empirical reporting delay distribution and simulating the generalized growth model, we estimated the effective reproduction number based on the discretized probability distribution of the generation interval | Secondary data analysis: The daily confirmed cases of COVID-19 in South Korea were extracted from publicly available sources | Effective reproduction number (Rt) from daily case incidence.  Intrinsic growth rate  The scaling of growth parameter.  The crude case fatality rate.  The implementation of social distancing measures | Four major clusters and estimated the reproduction number at 1.5 (95% CI: 1.4-1.6) were identified. In addition, the intrinsic growth rate was estimated at 0.6 (95% CI: 0.6, 0.7) and the scaling of growth parameter was estimated at 0.8 (95% CI: 0.7, 0.8), indicating sub-exponential growth dynamics of COVID-19. The crude case fatality rate is higher among males (1.1%) compared to females (0.4%) and increases with older age. The results indicate early sustained transmission of COVID-19 in South Korea and support the implementation of social distancing measures to rapidly control the outbreak.. |
| 17 | Burch & Bunt, (2020) | What are the effects of quarantine plus other measures for controlling the spread of COVID‐19? | Korea (only part of it) | Rapid review | Published | Quarantine plus other measures (such as work/school closures, isolation/social distancing, and travel restrictions) for controlling the spread of COVID‐19 | Literature review | Incidence: the transmission rate  (Forward transmission, Mortality, Resource use, Populations,  Intervention and comparator: Whit out data from Countries in study). | Incidence:  One South Korean modeling study estimated nearly 5 million COVID‐19 cases if no measures were taken. Implementing prevention and control measures could reduce the transmission rate by 90% or 99%, resulting in 0.5% or 0.4% of the 5 million predicted cases contracting COVID‐19. |

**References**

1. Sebastiani G, Massa M, Riboli E. Covid-19 epidemic in Italy: evolution, projections and impact of government measures. Eur J Epidemiol 2020;35(4):341–5. doi: 10.1007/s10654-020-00631-6
2. Tobías A. Evaluation of the lockdowns for the SARS-CoV-2 epidemic in Italy and Spain after one month follow up. Sci Total Environ 2020;725:138539. doi: 10.1016/j.scitotenv.2020.13853
3. Oksanen A, Kaakinen M, Latikka R, Savolainen I, Savela N, Koivula A. Regulation and Trust: 3-Month Follow-up Study on COVID-19 Mortality in 25 European Countries. JMIR Public Health Surveill 2020;6(2):e19218. doi: 10.2196/19218
4. Bruinen de Bruin Y, Lequarre AS, McCourt J, Clevestig P, Pigazzani F, Jeddi MZ, et al. Initial impacts of global risk mitigation measures taken during the combatting of the COVID-19 pandemic. Saf Sci 2020:104773. doi: 10.1016/j.ssci.2020.104773
5. Signorelli C, Scognamiglio T, Odone A. COVID-19 in Italy: impact of containment measures and prevalence estimates of infection in the general population. Acta Biomed 2020;91(3-S):175–9. doi: 10.23750/abm.v91i3-S.9511.
6. Nussbaumer-Streit B, Mayr V, Dobrescu AI, Chapman A, Persad E, Klerings I, et al. Quarantine alone or in combination with other public health measures to control COVID-19: a rapid review. Cochrane Database Syst Rev 2020;4:CD013574. doi: 10.1002/14651858.CD013574
7. Sjödin H, Wilder-Smith A, Osman S, Farooq Z, Rocklöv J. Only strict quarantine measures can curb the coronavirus disease (COVID-19) outbreak in Italy, 2020. Eurosurveillance 2020;25(13):pii=2000280. doi: 10.2807/1560-7917.ES.2020.25.13.2000280
8. Kim S, Seo YB, Jung E. Prediction of COVID-19 transmission dynamics using a mathematical model considering behavior changes in Korea. Epidemiol Health 2020;42:e2020026. doi: 10.4178/epih.e2020026.
9. Goldstein E, Lipsitch M. Temporal rise in the proportion of younger adults and older adolescents among coronavirus disease (COVID-19) cases following the introduction of physical distancing measures, Germany, March to April 2020. Euro Surveill 2020;25(17). doi: 10.2807/1560-7917.ES.2020.25.17.2000596
10. Gregori D, Azzolina D, Lanera C, Prosepe I, Destro N, Lorenzoni G, et al. A first estimation of the impact of public health actions against COVID-19 in Veneto (Italy). J Epidemiol Community Health 2020. doi: 10.1136/jech-2020-214209
11. Ambikapathy B, Krishnamurthy K. Mathematical modelling to assess the impact of lockdown on COVID 19 transmission in India: Model development and validation. JMIR Public Health Surveill 2020;6(2):e19368. doi: 10.2196/19368
12. Mandal S, Bhatnagar T, Arinaminpathy N, et al. Prudent public health intervention strategies to control the coronavirus disease 2019 transmission in India: A mathematical model-based approach. Indian J Med Res 2020;151(2 & 3):190–9. doi: 10.4103/ijmr.IJMR_504_20
13. Manchein C, Brugnago EL, da Silva RM, Mendes CFO, Beims MW. Strong correlations between power-law growth of COVID-19 in four continents and the inefficiency of soft quarantine strategies. Chaos 2020;30(4):041102. doi: 10.1063/5.0009454
14. Tomar A, Gupta N. Prediction for the spread of COVID-19 in India and effectiveness of preventive measures. Sci Total Environ 2020;728:138762. doi: 10.1016/j.scitotenv.2020.138762
15. COVID-19 National Emergency Response Center, Epidemiology and Case Management Team, Korea Centers for Disease Control and Prevention. Coronavirus Disease-19: The First 7,755 Cases in the Republic of Korea. Osong Public Health Res Perspect 2020;11(2):85-90. doi: 10.24171/j.phrp.2020.11.2.05
16. Shim E, Tariq A, Choi W, Lee Y, Chowell G. Transmission potential and severity of COVID-19 in South Korea. Int J Infect Dis 2020;93:339–44. doi: 10.1016/j.ijid.2020.03.031
17. Burch J, Bunt C. What are the effects of quarantine plus other measures for controlling the spread of COVID-19? Cochrane Clinical Answers 2020. doi: 10.1002/cca.3053
